# Stronger Pharmacological Cortisol Suppression and Anticipatory Cortisol Stress Response in Transient Global Amnesia

**DOI:** 10.3389/fnbeh.2015.00063

**Published:** 2015-03-09

**Authors:** Martin Griebe, Frauke Nees, Benjamin Gerber, Anne Ebert, Herta Flor, Oliver T. Wolf, Achim Gass, Michael G. Hennerici, Kristina Szabo

**Affiliations:** ^1^Department of Neurology, UniversitätsMedizin Mannheim, Heidelberg University, Mannheim, Germany; ^2^Department of Cognitive and Clinical Neuroscience, Central Institute of Mental Health, Heidelberg University, Mannheim, Germany; ^3^Department of Cognitive Psychology, Institute of Cognitive Neuroscience, Ruhr-University Bochum, Bochum, Germany

**Keywords:** transient global amnesia, cortisol, stress, hippocampus, memory

## Abstract

Transient global amnesia (TGA) is a disorder characterized by a sudden attack of severe anterograde memory disturbance that is frequently preceded by emotional or physical stress and resolves within 24 h. By using MRI following the acute episode in TGA patients, small lesions in the hippocampus have been observed. Hence, it has been hypothesized that the disorder is caused by a stress-related transient inhibition of memory formation in the hippocampus. To study the factors that may link stress and TGA, we measured the cortisol day-profile, the dexamethasone feedback inhibition and the effect of experimental exposure to stress on cortisol levels (using the socially evaluated cold pressor test and a control procedure) in 20 patients with a recent history of TGA and in 20 healthy controls. We used self-report scales of depression, anxiety and stress, and a detailed neuropsychological assessment to characterize our collective. We did not observe differences in mean cortisol levels in the cortisol day-profile between the two groups. After administration of low-dose dexamethasone, TGA patients showed significantly stronger cortisol suppression in the daytime profile compared to the control group (*p* = 0.027). The mean salivary cortisol level was significantly higher in the TGA group prior to and after the experimental stress exposure (*p* = 0.008 and 0.010 respectively), as well as prior to and after the control condition (*p* = 0.022 and 0.024, respectively). The TGA group had higher scores of depressive symptomatology (*p* = 0.021) and anxiety (*p* = 0.007), but the groups did not differ in the neuropsychological assessment. Our findings of a stronger pharmacological suppression and higher cortisol levels in anticipation of experimental stress in participants with a previous TGA indicate a hypersensitivity of the HPA axis. This suggests that an individual stress sensitivity might play a role in the pathophysiology of TGA.

## Introduction

Transient global amnesia (TGA) is characterized by a sudden attack of severe disturbance of anterograde episodic long-term memory disrupting the learning of novel episodic information (Mazzucchi and Parma, 1990; Jager et al., 2009) that resolves within 24 h (Hodges and Warlow, 1990). Several groups have suggested that the pathophysiological mechanisms leading to TGA may be similar to those of cerebral ischemia, epilepsy, or migraine (Frederiks, 1993). However, there is no definitive evidence supporting any of these mechanisms. More recently, a disturbance of venous hemodynamics has been hypothesized, but again without scientific data for such an underlying mechanism (Baracchini et al., 2012). Due to the clinical characteristics of the cognitive impairment during TGA, a transient dysfunction of the medial temporal lobes, especially of the hippocampus, has been postulated (Bartsch and Deuschl, 2010). In up to 80% of patients, diffusion-weighted MRI has been reported to show small lesions in the hippocampus 24–48 h after symptom onset (Gass et al., 1999; Sedlaczek et al., 2004; Bartsch et al., 2007). While this finding actually links the disorder to the CA-1 subfield of the hippocampus anatomically, the exact etiology of these lesions remains uncertain. Interestingly, physically or emotionally stressful episodes have been reported as precipitating events in up to 89% of TGA cases (Quinette et al., 2006). These observations have led to the implication that stress might play a role in the pathophysiology of TGA. More precisely, it has been hypothesized that TGA might be caused by a stress-related transient inhibition of memory formation in the hippocampus by means of a selective vulnerability of CA-1 neurons to metabolic stress (Bartsch and Deuschl, 2010). A recent study suggests that an elevated anxiety level may increase the susceptibility to psychological stress, which may facilitate the pathophysiological cascade in TGA (Dohring et al., 2014), however, the neuroendocrine response via the hypothalamic–pituitary–adrenal (HPA) axis has not been characterized.

To study the factors that may link psychological or physical stress to TGA on a neuroendocrine level, we measured the cortisol day-profile, dexamethasone feedback inhibition, and the effect of experimental exposure to stress on cortisol levels in 20 patients with a recent history of TGA and in 20 healthy controls. As experimental stress procedure, we used the socially evaluated cold pressor test (SECPT) during which subjects immerse their hand in ice water and are observed and videotaped by an unfamiliar experimenter. We used self-report scales of depression, anxiety, and stress and a detailed neuropsychological assessment to characterize our collective. We hypothesized that TGA patients exhibit a stronger HPA axis reactivity compared to the control subjects.

## Materials and Methods

### Participants

From our prospectively collected TGA database with 208 cases since 2001 fulfilling the established criteria (Hodges and Warlow, 1990), we contacted those 41 who had had a TGA in the years 2010 and 2011. Out of 26 patients willing to participate in the study, we included 20 right-handed TGA patients. Six patients were excluded due to meeting the following pre-specified exclusion criteria: left-handedness (*n* = 3), co-medication (corticosteroids, *n* = 2), and comorbidity interfering with the conduct of the trial (tremor, *n* = 1). We recruited 20 controls matched for age, sex, and education (see Table 1). The study received ethics approval, and all participants gave written informed consent. The study examinations were performed on three days: day 1 (in the morning) – neuropsychological evaluation, instruction for cortisol sample collection; day 2 and day 3 one-week apart at the same time of day (between 1 and 4 p.m.) – stress exposure or control condition in a randomized order.

**Table 1:** **Characteristics of the study population**.

	TGA	Controls	*p* value
Number	20	20	–
Age, years; mean (SD)	66.50 (±7.7)	66.55 (±7.0)	0.940
Sex, male; number	8	8	1.000
Formal education, years; median (range)	12.5 (8–19)	13 (8–17)	0.557
Participants with mental disorder^a^; number	6	1	**0.023**
MMSE, sum; median (range)	30 (28–30)	29.5 (28–30)	0.869
Time since TGA, months; mean (SD)	10.55 (11.15)	NA	–
Participants with DWI lesion; number	16	NA	–
Left hippocampus	4		
Right hippocampus	2		
Bilateral hippocampi	10		

*DWI, diffusion-weighted MRI; MMSE, mini mental state examination; SD, standard deviation*.

*Bold font indicates p < 0.05*.

*^a^Previously diagnosed depression or anxiety disorder*.

### HPA axis activity

Saliva samples were collected into Salivette tubes (Sarstedt, Nümbrecht, Germany), immediately after as well as 15, 30, 45, and 60 min after awakening (cortisol awakening response; Schmidt-Reinwald et al., 1999) and at 11 a.m., 1 p.m., 3 p.m., 6 p.m., 8 p.m., 10 p.m., and 11 p.m. (daytime profile). Salivary cortisol values were also sampled in an identical fashion on a second day following the administration of 0.5 mg dexamethasone at 11 p.m. (low-dose dexamethasone suppression test/dexamethasone challenge). Participants were instructed to refrain from smoking (all participants were non-smokers), drinking, or eating 10 min and brushing one’s teeth 5 min before each sampling. Participants completed a diary during the sampling period containing information on sleep duration, food intake, and daytime activities. Self-reports as well as an electronic monitoring device (MEMS Track Cap, Aardex, Switzerland) were employed to verify sample times. Cortisol data of two participants on day 1 (one TGA, one control) and three participants on day 2 (two TGA, one control) were excluded due to incongruence between self-report and electronic data. Saliva samples collected into Salivette tubes (Sarstedt, Nümbrecht, Germany) were kept at -20°C until analysis. Free cortisol levels in saliva were measured using a commercially available immunoassay (IBL, Hamburg, Germany) at the Institute of Biopsychology, Technische Universitaet Dresden, Dresden, Germany. Intra- and interassay variances were below 10%.

### Stress protocol

All participants were exposed to the SECPT (Schwabe et al., 2008). They were advised to immerse their right hand including the wrist in ice water (0–4°C) for 3 min or until they could no longer tolerate it. During the procedure, they were supervised by an unfamiliar person and informed that they were being videotaped for analysis of facial expression. All participants also underwent the control condition with warm water (35–37°C) during which they were not filmed. The order of the two procedures was randomized. Immediately after the SECPT and the control condition, participants were asked to rate how stressful, painful, and unpleasant the experiment had been (0 “not at all” to 100 “very”). Blood pressure (auscultatory method with stethoscope and aneroid sphygmomanometer), heart rate (manual palpation of radial artery), and salivary cortisol values were collected before, after 3 and after 15 min.

### Self-report scales of depression, anxiety, and stress

The German version of the CES-D (Hautzinger and Bailer, 1993) was used to measure depressive symptomatology. State and trait of anxiety were measured using the German version of the STAI (Laux et al., 1981) consisting of 40 questions on a self-report basis. The Trier inventory for chronic stress (TICS; Schulz and Schlotz, 1999) was employed to assess the level of chronic psychosocial stress arising from environmental and internal demands in several domains in a defined time frame.

### Neuropsychological assessment

All participants underwent a standardized neuropsychological assessment of the following domains: global cognitive function [Mini Mental State Examination (Folstein et al., 1975)], semantic memory [recognition vocabulary from the Achievement Measure System (Horn, 1983)], semantic word fluency [Regensburg Vocabulary Test (Aschenbrenner et al., 2001)], reaction time [alertness assessment from the Test for Attentional Performance (Zimmermann and Fimm, 2012)], executive functions [lexical fluency from the Regensburg Vocabulary Test (Aschenbrenner et al., 2001)]; selective attention in Go–Nogo of the Achievement Measure System (Zimmermann and Fimm, 2012), visuo-spatial functions [mental rotation and figure-ground perception of the Achievement Measure System (Horn, 1983)], verbal and non-verbal short-term memory [digit and block span forwards of the Revised Wechsler Memory Scale (Härting et al., 2000)], verbal and non-verbal working memory [digit and block span backwards of the Revised Wechsler Memory Scale (Härting et al., 2000)], verbal episodic memory [Auditory Verbal Learning Test (Helmstaedter and Lux, 2001)], and visual episodic memory [route learning and retrieval of the Visual and Verbal Memory Test (Schellig and Schächtele, 2009); pattern recognition of the (CANTABeclipse, 2006)].

### Statistical analysis

Neuropsychological test results and self-ratings were analyzed by independent samples *t*-tests or Mann–Whitney-*U*-tests as appropriate. Group differences in the circadian profiles of cortisol concentrations between TGA patients and controls were calculated by one-way repeated measures ANOVA with 12 within-group levels. To compare cortisol levels for each sampling point, we used independent samples *t*-tests. Area under the curve with respect to ground (AUCg) and area under the curve with respect to increase (AUCi) were calculated for both circadian profiles of cortisol and compared between groups by independent samples *t*-tests (Pruessner et al., 2003). Group differences in cortisol, blood pressure, and heart rate responses to SECPT and the control procedure were calculated by one-way repeated measures ANOVA with three within-group levels. Thereafter, data for each of the three sampling points were compared by independent samples *t*-tests and alterations in the time course were calculated by dependent samples *t*-tests. To compare duration of SECPT between groups, we performed independent samples *t*-tests, to compare self-ratings of reactive stress and pain, Mann–Whitney-*U*-tests were performed. Pearson’s correlation coefficient was calculated to test for a linear correlation between cortisol levels before the SECPT or the control procedure and the results of CES-D, STAI, and TICS, as well as between the stress reactivity (difference last cortisol level after SECPT or the control procedure and cortisol level before) and the results of CES-D, STAI, and TICS.

## Results

### Study population

**Figure 1:**
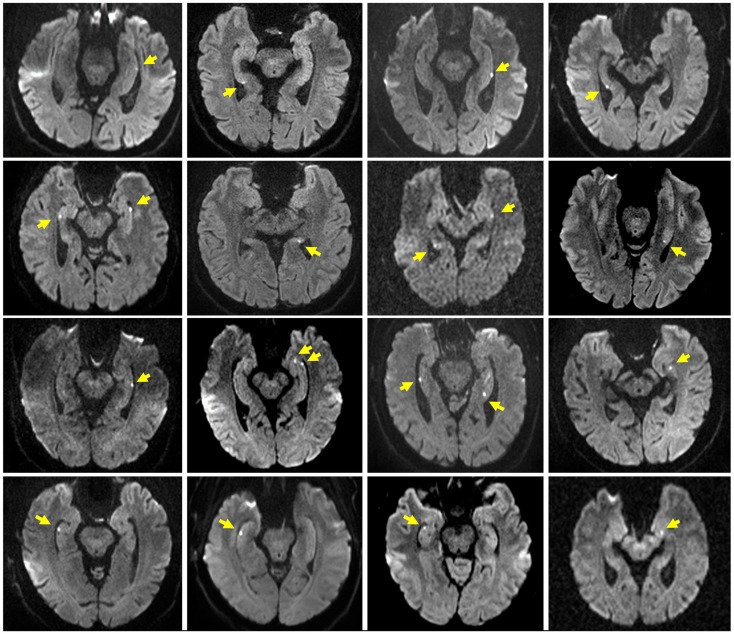
**Hippocampal MRI in TGA patients**. Representative diffusion-weighted MR images after the acute episode of TGA in 16 of the 20 patients. Slice positioning parallel to the long axis of the hippocampus. Yellow arrows indicate the hippocampal lesions.

Table 1 summarizes the characteristics of the study population. The TGA and control group did not differ significantly with respect to age, sex, education, and MMSE score. Patients with a former TGA had a significantly (*p* = 0.023) higher incidence of a previously diagnosed mental disorder (TGA: depression, *n* = 5, anxiety disorder, *n* = 1; controls: depression, *n* = 1). Sixteen patients showed a hippocampal diffusion-weighted MRI lesion after the TGA episode (see Figure 1).

### Cortisol status

**Figure 2:**
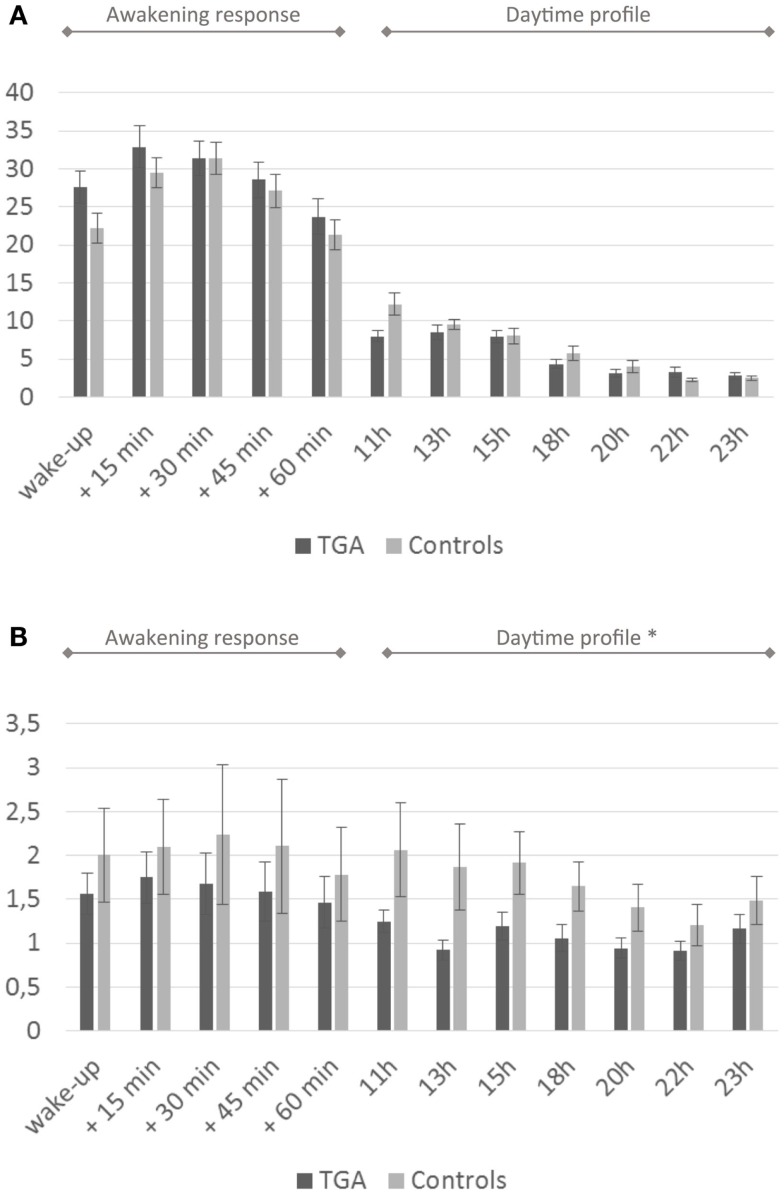
**Cortisol profiles**. **(A)** Daytime profile of salivary cortisol levels. Twenty-four-hour cortisol profile in 20 participants with a history of TGA and 20 age and sex matched controls. Although the cortisol awakening response is higher in the TGA group, this is not significant (*p* = 0.140). **(B)** Salivary cortisol levels after dexamethasone challenge. Twenty-four-hour cortisol profile in 20 participants with a history of TGA and 20 age and sex matched controls following the administration of 0.5 mg dexamethasone at 11 p.m. on the day before. TGA patients show a significantly stronger cortisol suppression in the daytime profile compared to the control group (*p* = 0.027). Data are presented as the mean (in nmol/l) at predefined sample times (awakening, +15, +30, +45, +60 min, thereafter daytime profile) across subjects. * indicates significant group difference.

We found no significant differences in mean cortisol levels during the awakening response or in the daytime cortisol profile between the two groups. After administration of low-dose dexamethasone, the TGA patients showed significantly stronger cortisol suppression in the daytime profile compared to the control group (*p* = 0.027). For details see Figure 2 and Table 2.

**Table 2:** **Cortisol profiles**.

	TGA	Controls	*p* value
**Cortisol status**
Cortisol awakening response, AUCg; nmol/l h; mean (SEM)	118.40 (±7.81)	109.64 (±7.12)	0.213
Cortisol awakening response, AUCi; nmol/l h; mean (SEM)	8.11 (±8.66)	20.91 (±7.38)	0.140
Cortisol daytime profile, AUCg; nmol/l h; mean (SEM)	68.03 (±4.34)	78.02 (±6.27)	0.105
DST awakening response, AUCg; nmol/l h; mean (SEM)	6.53 (±1.11)	2.85 (±2.61)	0.273
DST awakening response, AUCi; nmol/l h; mean (SEM)	0.28 (±0.95)	0.33 (±0.89)	0.486
DST daytime profile, AUCg; nmol/l h; mean (SEM)	12.53 (±1.20)	20.06 (±3.41)	**0.027**
**Socially evaluated cold pressor test**
Salivary cortisol level before SECPT, nmol/l; mean (SEM)	11.28 (±1.44)	7.12 (±0.75)	**0.008**
Salivary cortisol level after SECPT, nmol/l; mean (SEM)	11.81 (±1.34)	7.92 (±0.86)	**0.010**
Salivary cortisol level after 15 min, nmol/l; mean (SEM)	13.72 (±2.08)	12.25 (±1.41)	0.281

*AUCg, area under the curve with respect to ground; AUCi, area under the curve with respect to increase; DST, dexamethasone suppression test; SEM, standard error of the mean*.

*Bold font indicates p < 0.05*.

### Socially evaluated cold pressor test

None of the participants suffered any adverse effects (including headaches) after the stress exposure. The mean salivary cortisol level was significantly higher in participants with a history of TGA already prior to (*p* = 0.008) and immediately after the SECPT (*p* = 0.010) than in those without (see Table 2). After an additional 15 min, salivary cortisol values were not different between the TGA group and those without TGA (*p* = 0.281). We found a significant increase of cortisol in the control group between the first and last measurements (before and 15 min after SECPT; *p* = 0.002), but not in the TGA group (*p* = 0.171).

Interestingly, during the control condition, we made a similar observation: The mean salivary cortisol level was significantly higher in participants with a history of TGA already prior to (*p* = 0.022) and immediately after the 3 min warm water procedure (*p* = 0.024) than in the control group. After an additional 15 min, salivary cortisol values were not significantly different between the groups (*p* = 0.134). During this experiment, the TGA group showed a decrease in cortisol levels from the first to the third measurement. However, this decrease was not statistically significant (*p* = 0.099).

**Figure 3:**
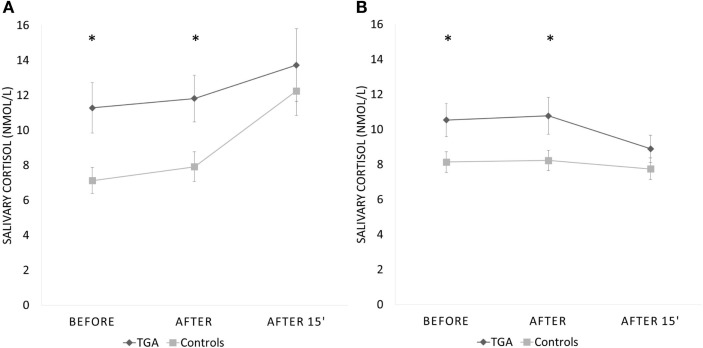
**Cortisol response to experimental stressor**. Salivary cortisol response (in nmol/l) to the socially evaluated cold pressor test **(A)** and the control procedure with warm water **(B)** in 20 participants with a history of TGA and 20 normal controls. Data are presented as mean ± standard error of the mean. *Indicates significant group difference.

Repeated measures analysis for interaction between groups missed significance (*p* = 0.082). Figure 3 shows the change across the three time points during both procedures for participants with a history of TGA and the controls. Eight participants (six TGA and two controls) did not immerse their hand for the full 3 min in ice water (TGA group mean duration = 146 s, range: 28–180 s; control group mean duration = 169 s, range 50–180; *p* = 0.065). The TGA group perceived the SECPT as significantly more stressful (*p* = 0.036) but not as more painful (*p* = 0.130). There were no significant differences in increase of systolic or diastolic blood pressure or heart rate between the two groups during the SECPT.

### Self-report scales and neuropsychological assessment

The TGA group had higher levels of depressive symptomatology and higher scores of anxiety compared with the control group as assessed by the CES-D (*p* = 0.021) and the STAI (*p* = 0.007), respectively (see Table 3). There was no significant difference between the two groups for chronic stress as measured by the TICS (*p* = 0.134).

**Table 3:** **Personality trait evaluation**.

	TGA	Controls	*p* value
**Self-report scales**
CES-D, sum; mean (SEM)	12.3 (±1.19)	8.5 (±1.36)	**0.021**
STAI sum; mean (SEM)	39.3 (±1.96)	31.0 (±2.07)	**0.007**
TICS sum; mean (SEM)	67.15 (±3.77)	59.2 (±3.08)	0.134

*CES-D, Center for Epidemiological Studies Depression Scale; SEM, standard error of the mean; STAI, state-trait anxiety scale; TICS, trier inventory for chronic stress*.

*Bold font indicates p < 0.05*.

There were no significant differences in the results of the neuropsychological assessment between the two groups in any of the used measures: semantic memory (*p* = 0.686), semantic word fluency (*p* = 0.929), reaction time (*p* = 0.326), executive functions (lexical fluency *p* = 0.884; selective attention speed *p* = 0.146; false positive reactions *p* = 0.057), visuo-spatial functions (mental rotation *p* = 0.798 and figure-ground perception *p* = 0.949), verbal and non-verbal short-term memory (digit *p* = 0.774 and block span forward *p* = 0.704), verbal and non-verbal working memory (digit *p* = 0.848 and block span backward *p* = 0.842), verbal episodic memory (learning, sum of trials 1–5, *p* = 0.434; retroactive interference trials 5 and 6, *p* = 0.200; late recall loss, trials 5–7, *p* = 0.125; recognition, hits – false positives *p* = 0.628), and visual episodic memory (route learning *p* = 0.836 and retrieval *p* = 0.926; immediate recognition *p* = 0.150; delayed recognition *p* = 0.927).

### Correlations with cortisol response

We found no correlation between the first cortisol value before the SECPT or before the control procedure and the CES-D, STAI, or TICS scores in either group. Similarly, there was no correlation between the CES-D, STAI, or TICS score and the stress reactivity, defined as the difference between the last and the first cortisol measurement of the SECPT or the control procedure.

## Discussion

In this study, we aimed to investigate the possible role of stress in TGA. We observed a significantly stronger daytime suppression of cortisol secretion in response to low-dose dexamethasone and higher levels of cortisol in TGA patients before and after an experimental stress condition but also before and after the control procedure.

The possible role of certain personality traits – making individuals susceptible to stress – has been reported in TGA patients. Certain aspects of personality have been suggested to be more common in TGA: Inzitari et al. (1997) found phobic personality traits in 82% of 51 TGA patients. Also, depressive symptoms and comorbidities with mental disorder have been claimed to be more common in TGA (Neri et al., 1995; Pantoni et al., 2005). In their series of 142 patients, Quinette et al. (2006) reported a high frequency of psychological and emotional instability suggesting that TGA patients might be particularly vulnerable to psychological stress. Similarly, a recent study came to the conclusion that TGA patients might cope with stress less efficiently and less constructively than controls (Dohring et al., 2014). The authors report that patients with a stress-related precipitating event had a higher level of anxiety as measured by self-rating questionnaires in comparison to patients without such an event and controls. Our study confirms previous observations of higher levels of depressive symptomatology, higher scores of anxiety, as well as a higher prevalence of preexisting psychiatric comorbidities in TGA patients. Concerning the reversibility of memory deficits in TGA, many studies have shown complete recovery of memory functioning several months after an episode of TGA (Kritchevsky et al., 1988; Kritchevsky and Squire, 1989; Faglioni et al., 1992; Quinette et al., 2003; Bartsch et al., 2006; Uttner et al., 2007). Similarly, we found no significant differences in the results of the neuropsychological assessment between groups.

Already in 1964, the presence of certain events occurring immediately before the attack – such as swimming in cold water, taking hot showers, pain, and sexual intercourse – was described (Fisher and Adams, 1964). As more recently analyzed, in up to 90% (32–89.1%) of reported TGA cases, a precipitating event – mainly described as physical, emotional, or behavioral stress – has been reported (Quinette et al., 2006). In addition to those mentioned above, typical such events include stressful medical examinations, arguments, legal proceedings, funerals, exhausting physical work, and celebrations (Schmidtke and Ehmsen, 1998; Quinette et al., 2006; Griebe et al., 2015). In addition to events immediately before the attack, remote factors preceding TGA by days or weeks, for example, anxiety, exhaustion, and financial worries have also been described (Quinette et al., 2006). We used the SECPT, an experimental stress procedure that has been shown to lead to an HPA axis system activation (Schwabe et al., 2008). This stressor seems very appropriate, as immersion in cold water has not only been reported in case series of TGA leading to the term “amnesia by the seaside” (Martin, 1970), but has also been reported to lead to an amnestic episode in an experimental setting (Castellani et al., 1998). In our study, TGA patients exhibited elevated cortisol levels already before and immediately after both the SECPT and the control condition compared to the control subjects. Due to the physiologically slow HPA axis mediated cortisol response, the values obtained at these two timepoints cannot be regarded as the reactive cortisol response, but rather as an anticipatory cortisol response (Engert et al., 2013). Since the procedures were scheduled in a blinded and randomized order, we assume that TGA patients reacted to the upcoming and potentially stressful experiment. The anticipatory cortisol response can be interpreted as a manifestation of increased subjective stress sensitivity. This is in line with observations in posttraumatic stress disorder (Bremner et al., 2003), phobia (Alpers et al., 2003), and alexithymia (de Timary et al., 2008). The higher self-reported anxiety in our patients strengthens this possible mechanism. In contrast, the reactive cortisol response 15 min after the SECPT was not significantly different between the groups.

Recent research suggests that the interaction of genetic and cognitive or emotional factors in which glucocorticoid hormones and receptors play a crucial role might be responsible for the differences of coping with and adaptation to stress in individuals (Oitzl et al., 2010). In selected mental disorders, including major depressive disorder, posttraumatic stress disorder, and borderline personality disorder, the interaction of stress, the hippocampus, and memory function is well characterized by alterations of the HPA axis and hippocampal dysfunctions (Wingenfeld and Wolf, 2014). We observed an alteration of the HPA axis regulation with a stronger suppression after dexamethasone pre-treatment in our collective of TGA patients, a pattern consistently reported in patients with posttraumatic stress disorder, opposed to non-suppression in patients with major depressive disorder (Handwerger, 2009). Analogous to previous observations in posttraumatic stress disorder (Yehuda et al., 2004) we assume that the depressive status in the TGA group did not impact the HPA axis activity. Last but not least the intact cortisol awakening response is in sharp contrast to the absent cortisol awakening response occurring in patients with global (non-transient) amnesia (Wolf et al., 2005) and in patients with hippocampal damage (Buchanan et al., 2004).

Our findings in participants with a previous TGA indicate a hypersensitivity of the HPA axis with stronger pharmacological suppression and higher cortisol levels in anticipation of experimental stress. This neuroendocrine pattern shares similarities with stress and trauma associated psychiatric disorders. In TGA, the enhanced stress sensitivity may result in the pathophysiological activation of metabolic responses affecting the function of the hippocampus on a cellular level. Activation of the HPA axis during accidental or experimentally induced stress is known to lead to an elevation of glucocorticoid hormone levels. High glucocorticoid levels in turn have been shown to increase neuronal vulnerability in the hippocampus, to induce a decrease in regional cerebral blood flow in the mesial temporal lobe, and to have a negative effect on cognition and memory (Lupien and Lepage, 2001). Our findings support the possible role of a stress-induced cascade of steroid-mediated glutamatergic cytotoxicity, effecting the structural integrity of CA-1 neurons in the hippocampus (Bartsch and Deuschl, 2010).

For critical interpretation of our data, we assessed the possible effect of the identified personality traits of the TGA group on the cortisol values during experimental stress exposure but found no correlation between either the first cortisol value or the stress reactivity and the CES-D, STAI or TICS scores. Previous studies found no close relationship between personality factors and stress-induced salivary cortisol increases (Kudielka et al., 2009). However, we cannot differentiate whether our findings are a prerequisite condition for or possibly the consequence of a TGA episode.

In conclusion, our findings reveal a hypersensitivity of the HPA axis and suggest that an individual stress sensitivity might play a role in the pathophysiology of TGA.

## Author Contributions

MG, FN, and KS contributed to study conception and design, data collection and analysis, interpretation, drafting, and revising the manuscript. BG and AE contributed to data collection and analysis and revision of the manuscript. HF contributed to study conception and design, data analysis, interpretation, and revision of the manuscript. OW contributed to data analysis, interpretation, and revision of the manuscript. AG and MH contributed to study conception and design, data analysis, and revision of the manuscript. All authors have approved the final manuscript version to be published and have agreed to be accountable for all aspects of the work in ensuring that questions related to the accuracy or integrity of any part of the work are appropriately investigated and resolved.

## Conflict of Interest Statement

Frauke Nees and Kristina Szabo have received funding for this study from the Deutsche Forschungsgemeinschaft (DFG); project C07 of the Collaborative Research Center 636 “Learning, memory and brain plasticity: implications for psychopathology.” Martin Griebe, Benjamin Gerber, Anne Ebert, Herta Flor, Oliver T. Wolf, Achim Gass, and Michael G. Hennerici declare that the research was conducted in the absence of any commercial or financial relationships that could be construed as a potential conflict of interest.

## Abbreviations

AUCg, area under the curve with respect to ground; AUCi, area under the curve with respect to increase; CES-D, center for epidemiological studies depression scale; HPA axis, hypothalamic–pituitary–adrenal axis; SECPT, socially evaluated cold pressor test; STAI, state-trait anxiety inventory; TGA, transient global amnesia; TICS, trier inventory for chronic stress.
